# Serine/threonine kinases 31(STK31) may be a novel cellular target gene for the HPV16 oncogene E7 with potential as a DNA hypomethylation biomarker in cervical cancer

**DOI:** 10.1186/s12985-016-0515-5

**Published:** 2016-04-05

**Authors:** Fu-Fen Yin, Ning Wang, Xiao-Ning Bi, Xiao Yu, Xiao-Hui Xu, You-Lin Wang, Cheng-Quan Zhao, Bing Luo, Yan-Kui Wang

**Affiliations:** Department of Obstetrics and Gynecology, Affiliate Hospital of Qingdao University, Qingdao, China; Department of Obstetrics and Gynecology, Peking Union Medical College Hospital, Chinese Academy of Medical Sciences and Peking Union Medical College, Beijing, China; Department of Urology, Shanghai Institute of Andrology, Ren Ji Hospital, School of Medicine, Shanghai Jiao Tong University, Shanghai, China; Department of Pathology, Magee-Womens Hospital, University of Pittsburgh Medical Center, Pittsburgh, PA USA; Department of Medical Microbiology, Qingdao University Medical College, Qingdao, 266021 China

**Keywords:** Cervical cancer, STK31, Human papillomavirus, DNA methylation, 5-aza-2′deoxycytidine, Biomarker

## Abstract

**Background:**

Cervical cancer (CC) is a leading cause of mortality in females, especially in developing countries. The two viral oncoproteins E6 and E7 mediate the oncogenic activities of high-risk human papillomavirus (hrHPV), and hrHPV, especially HPV16 or/and HPV18 (HPV16/18) play critical roles in CC through different pathways. STK31 gene of which the expression has been proven to be regulated by the methylation status of its promoter, is one of the novel cancer/testis (CT) genes and plays important roles in human cancers. Reasearches have indicated that viral infection is correlated to the methylation statuses of some genes. Herein, we detected methylation status of the STK31 gene in cervical tumors and explored its interaction with HPV16 or/and HPV18 (HPV16/18) infection.

**Methods:**

Bisulfite genomic sequencing PCR (BGS) combined with TA clone, methylation-specific PCR (MSP) were used to analyze methylation statuses of the STK31 gene promoter/exon 1 region in HPV16/18-positive, HPV-negative CC cell lines; ectopically expressed HPV16 E6, -E7, and -E6/E7 CC cells; normal cervical tissues and cervical tumor tissues of different stages. The mRNA and protein expressions of STK31 were detected by RT-PCR and western blotting.

**Results:**

The STK31 gene promoter/exon 1 was hypomethylated in the HPV16/18-positive cell lines HeLa, SiHa and CaSki, and the mRNA and protein expression were detected. In contrast, the STK31 gene exhibited hypermethylation and silenced expression in the HPV-negative CC cells C33A and HT-3. Compared with the primary HPV-negative CC cell lines, the STK31 methylation was downregulated, and STK31 expression was induced in the HPV16E7/E67 transfected cells. The methylation statuses and expressions of STK31 were verified in the cervical tumor samples at different stages. Additionally, chemotherapy treatment may influence STK31 expression by regulating its methylation status.

**Conclusions:**

STK31 may be a novel cellular target gene for the HPV16 oncogeneE7. The HPV16 oncogene E7 may affect STK31 expression through a methylation-mediated mechanism. The aberrant methylation of the STK31 promoter/exon 1 region may be a precursor of human cervical carcinogenesis and a potential DNA aberrant methylation biomarker of conditions ranging from precancerous disease to invasive cancer.

**Electronic supplementary material:**

The online version of this article (doi:10.1186/s12985-016-0515-5) contains supplementary material, which is available to authorized users.

## Background

CC is a leading cause of mortality in females, especially in developing countries [1]. The two viral oncoproteins E6 and E7 mediate the oncogenic activities of high-risk human papillomavirus (hrHPV), especially HPV16 or/and HPV18 (HPV16/18), which have been demonstrated to play critical roles in CC through different pathways [2, 3]. HrHPV alone is necessary but insufficient for cervical carcinogenesis; only a small proportion of HPV-infected patients develop invasive CC, and the majority remain subclinical or exhibit only precursor lesions [4, 5]. Though there has been some of molecular and cytological tests, some of which are commercially available (e.g. LCT, HC2 and CINTec Plus), that provide helpful data to determine the potential risk of HPV-infected patients to develop cancer, it is still important to detect novel biomarkers that are able to identify the women who are at risk of developing CC from among those who are persistently hr-HPV infected.

Research has indicated that epigenetic abnormalities, particularly aberrant methylation changes, play causative roles in tumorigenesis [6, 7]. Though studies have been focused on aberrant methylation of many tumor supressor genes, some tumor genes also have been demonstrated to be associated with tumorigenesis [7–9]. STK31 is one of the novel cancer/testis (CT) genes [10, 11], and the expression of STK31 has been proven to be regulated by the methylation status of its promoter [12]. The results have demonstrated that the STK31 gene play crucial roles in human cancers. These roles may involve cell cycle regulation; the overexpression of STK31 increases cell migration and invasiveness, whereas the depletion of STK31 induces apoptosis [13]. STK31 has also been revealed to maintain the undifferentiated state of colon cancer cells [12]. However, the biological function of STK31 and the potentially associated epigenetic mechanism have not yet been investigated in CC.

In this study, we investigated the influences of HPV16 E6 and E7 on the expression and methylation status of the STK31 gene in CC cell lines. Additionally, we tested the methylation levels and expression changes in STK31 in HPV16/18-positive and HPV-negative cervical lesions in different stages. We also explored the correlations between chemotherapy and STK31 gene methylation changes in CC cases.

## Results

### HPV16 E7 and E6/E7 oncoproteins induced or influenced STK31 gene expression in CC cells through regulating the methylation status of STK31

**Fig. 1:**
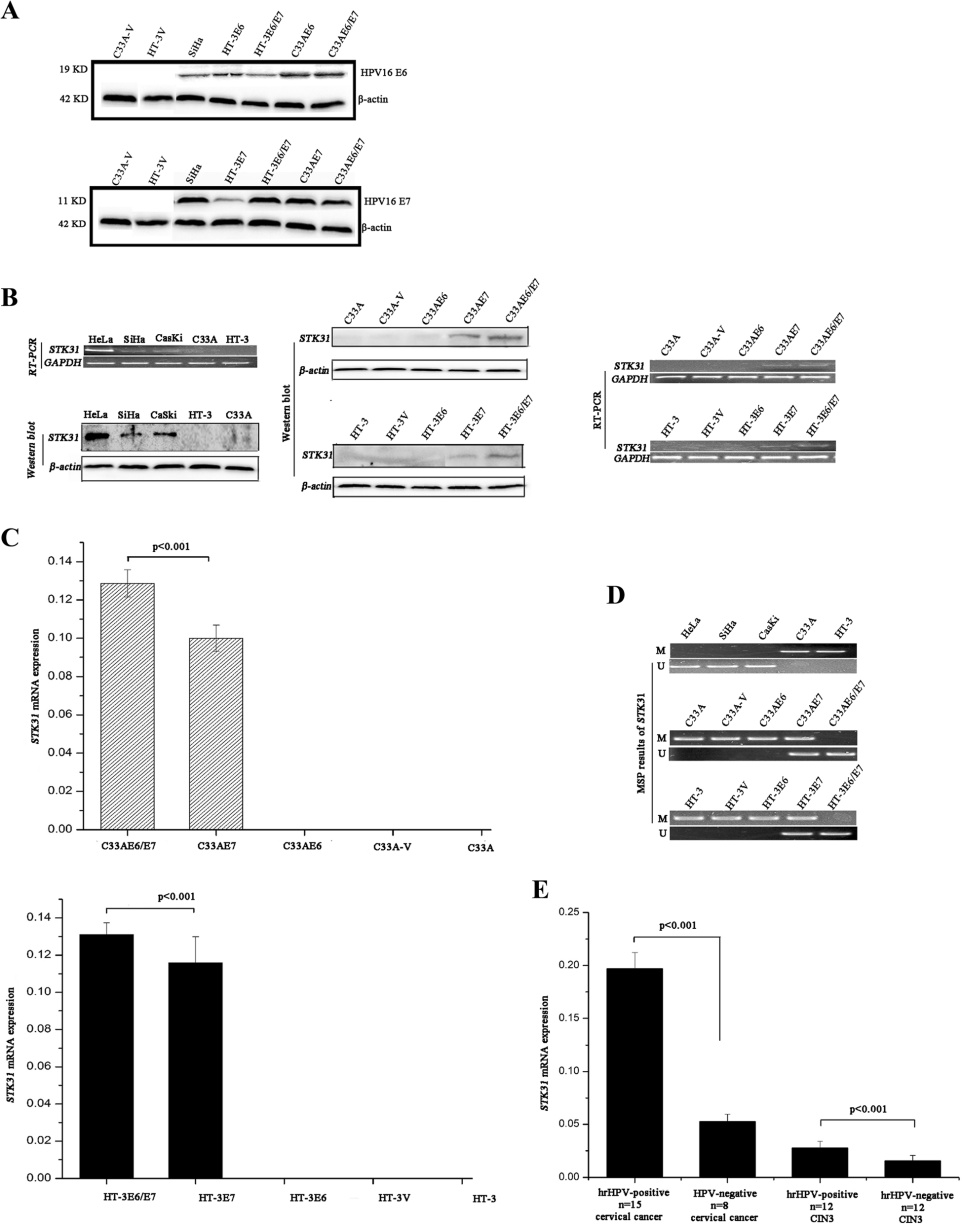
HPV16 E7 and E6/E7 induced or influenced STK31 expression through an epigenetic mechanism and STK31 expression varied with the development of cervical tumors. **a** The transfection efficiency of HPV16 E6, E7及E6/E7 was tested by Western blot. SiHa: positive control. **b** RT-PCR and western blotting results regarding STK31 expression in the HPV16/18-positive cervical cell lines HeLa, SiHa, and CaSki and the HPV-negative CC cell lines C33A and HT-3; results of RT-PCR and western blotting results indicating that STK31 was re-expressed in the HPV16 E6 or/and E7 oncogene-transfected cells except C33AE6 and HT-3E6. **c** qPCR results showing that the STK31 expression levels in the C33AE6/E7 and HT-3E6/E7 cells were greater than those in the C33AE7 and HT-3E7 cells, respectively. **d** The MSP results were consistent with the BGS results and indicated that the STK31 promoter/exon 1 was hypermethylated in the C33A and HT-3 cell lines and hypomethylated in the HeLa, SiHa, and CaSki cell lines. Decreased methylation statuses were detected in the C33AE7, C33AE6/E7, HT-3E7, and HT-3E6/E7 cell lines, and no methylation changes were observed in the C33AE6 and HT-3E6 cells compared with the C33A-V and HT-3 V cells, respectively. **e** The expression of STK31 was higher in the HPV16/18-positive cases than in the HPV-negative cases of CC and CIN3 (*p* < 0.001)

The transfection efficiency was tested by western blotting, which revealed that the transfected cells successfully expressed the E6, -E7, or -E6/E7 proteins (Fig. 1a). The RT-PCR and western blotting results (Fig. 1b) revealed that STK31 expression was present in the HPV16/18-positive cervical cell lines HeLa, SiHa, and CaSki cells but silenced in the HPV-negative CC cell lines C33A and HT-3 cells. Among the HPV16 E6- and/or E7-transfected cell lines, STK31 expression was detected in the C33AE7, C33AE6/E7, HT-3E7 and HT-3E6/E7 cells but not in the C33A-V, C33AE6, HT-3 V or HT-3E6 cell lines (Fig. 1b). Furthermore, the qPCR results indicated that the STK31 expressions in the C33AE6/E7 and HT-3E6/E7 cells were greater than those in the C33AE7 and HT-3E7 cells, respectively (Fig. 1c).

To investigate whether STK31 expression could be regulated by its promoter/exon 1 region, we tested the methylation status of STK31 promoter/exon 1 region. The analyzed STK31 gene sequence contains 37 CpG sites in its promoter/exon 1 region. The BGS statistics (Additional file 1: Figure S1D-1) of the STK31 promoter/exon 1 revealed that the CpG islands of STK31 were hypomethylated in the HeLa, SiHa and CaSki cell lines (48.2, 50.0, and 44.6 %, respectively), whereas the CpG islands of the STK31 gene were nearly completely methylated in the two HPV-negative cell lines (i.e., the C33A and HT-3 cells; 95.5 and 97.7 %, respectively). Among the cells that ectopically expressed HPV16 oncoproteins, the CpG methylation frequencies of STK31 in C33AE7, C33AE6/E7, HT-3E7, and HT-3E6/E7 (53.2, 46.8, 60.0, and 49.1 %, respectively) were decreased compared with those in the C33A-V and HT-3 V cells (95.5 and 96.8 %, respectively), whereas no significant demethylation changes were observed in the C33AE6 or HT-3E6 cells (90.1 and 98.2 %, respectively). The CpG methylation rates of STK31 gene in the C33A-V and HT-3 V mock vector-transfected cells (95.5 and 96.8 %, respectively) remained almost unchanged relative to the C33A and HT-3 cells (95.5, 98.2 %, respectively). The MSP results (Fig. 1d) were consistent with those from the BGS assay.

### STK31 gene expression was higher in the HPV16/18 -positive cervical tumors than the HPV-negative tumors

To further determine whether the expression of STK31 was associated with HPV16/18 infection, the expressions of STK31 in the HPV16/18-positive and HPV-negative cases of CC and HPV16/18-positive and HPV-negative cases of cervical intraepithelial neoplasia3 (CIN3) were studied with qPCR. The expression of STK31 was higher in the HPV16/18-positive cervical tissues among CC tissues and CIN3 specimens than those in the HPV-negative ones (*p* < 0.001, Fig. 1e). The STK31 expressions of cervical tissues seem to be inconsistent with the cell lines, because Fig. 1e, mean STK31 expression values of the HPV-negative CC tissues and the CIN3 specimens are presented, while the RT-PCR in Fig. 1b shows no STK31 mRNA and protein at all in HPV-negative cancer cells C33A and HT-3. Reasons for it are that STK31 mRNA does express a low level in most of HPV-negative clinical CC and CIN3 samples, but it just hasn’t detected in C33A and HT-3 cells.

### The STK31 methylation statuses varied consistently with the development of cervical tumors

**Fig. 2:**
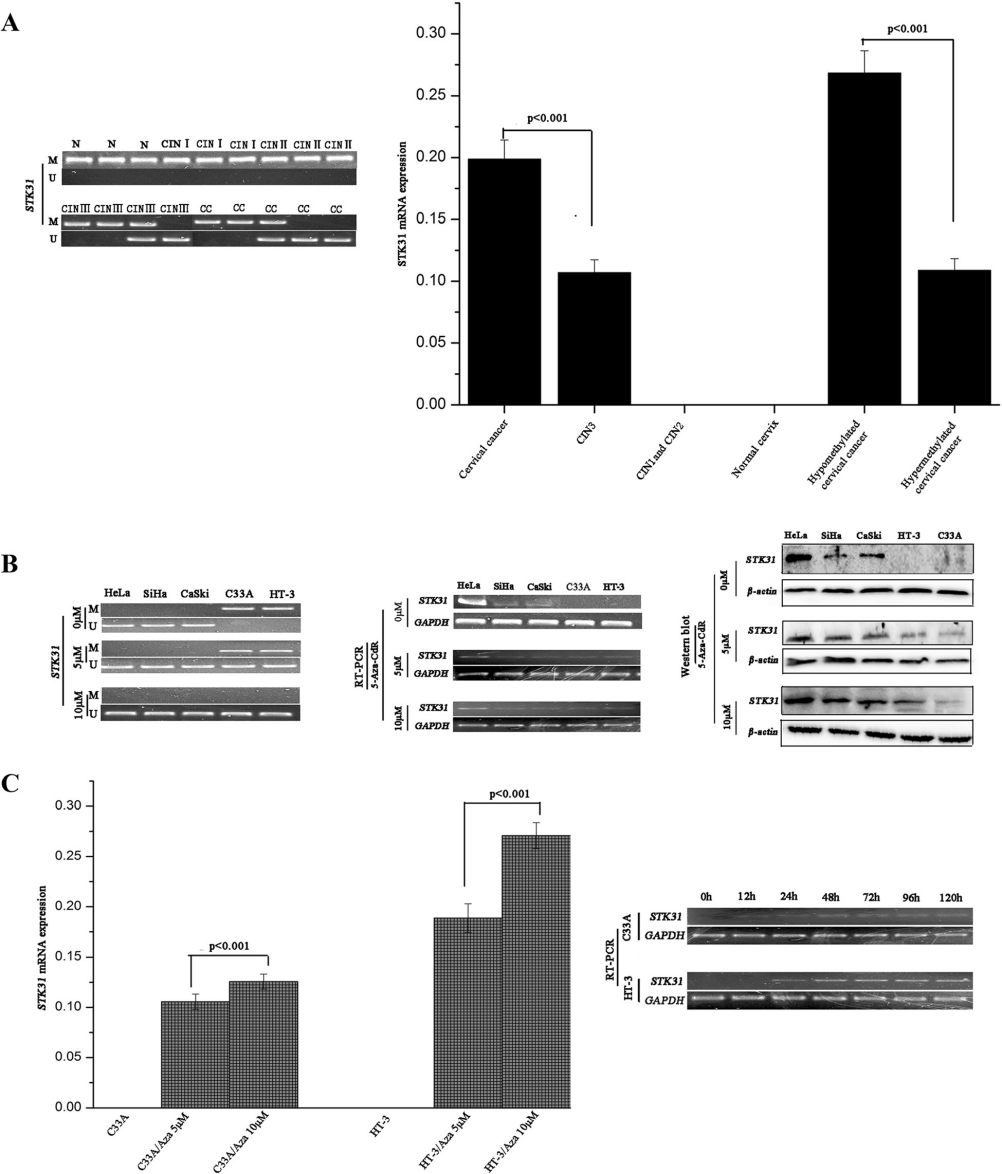
STK31 expression was different in the development of cervical lesions and the expression was regulated by its methylation status. **a** MSP and qPCR revealed that methylation status and expression of STK31 gene were different in different stages of cervical lesions. **b** Results of MSP and western blotting showed that demethylating agent 5-aza-dC induced STK31 expression of C33A and HT-3 cells by down-regulating its promoter/exon1 methylation status. **c** The effects of 5-aza-dC on STK31 gene and was concentration and time depedent

To determine the potential roles of STK31 methylation changes in cervical carcinogenesis, we analyzed the methylation statuses of STK31 in benign cervical tissues and various cervical lesions. MSP analyses were conducted in 42 HPV16/18-positive normal cervical samples, 20 cases of HPV16/18-positive CIN1, 20 cases of HPV16/18-positive CIN2, 48 cases of HPV16/18-positive CIN3, and 148 HPV16/18-positive CC tissues. Hypermethylation of STK31 was detected in all of the normal cervical tissues and the CIN1and CIN2 tissues. However, hypomethylation of STK3 was observed in 75.0 % of the CIN3 cases (36/48) and 22.3 % of the CC cases (33/148). Examples MSP analyses are presented in Fig. 2a.

### STK31 expression varied with the development of cervical tumors, and the expression was regulated by its promoter/exon 1 methylation status

Regarding the clinical samples, the statistical tests of the qPCR data (Fig. 2a) demonstrated that the expression of STK31 in the HPV16/18-positive CC specimens (0.1987 ± 0.0156, mean ± SD) was upregulated compared to that in the HPV16/18-positive CIN3 samples (0.1069 ± 0.0105, mean ± SD; *p* < 0.05), and no expression was detected in the HPV16/18-positive normal cervical tissue, HPV16/18-positive CIN1 or HPV16/18-positive CIN2 tissues. The qPCR results (Fig. 2a) revealed that the expression of STK31 was greater in the HPV16/18-positive CC samples with hypomethylation (0.2683 ± 0.0180, mean ± SD) than those HPV16/18-positive CC samples with hypermethylation (0.1087 ± 0.0095, mean ± SD; *p* < 0.05).

To further validate whether STK31 expression is regulated by its methylation status, HeLa, SiHa, CaSki, C33A and HT-3 cell lines were treated with 5 μM and 10 μM 5-aza-dC for 4 days. BGS analysis (Additional file 2: Figure S2B-1) revealed that demethylation was induced in the previously hypermethylated C33A and HT-3 cell lines, while no significant methylation changes were detected in the HeLa, SiHa, or CaSki cells following 5-aza-dC treatment. The MSP results (Fig. 2b) agreed with the BGS results. STK31 transcript and protein re-expression was detected in the C33A and HT-3 cells, but no significant expression changes were observed in the HeLa, SiHa, or CaSki cells following the 5-aza-dC treatment (Fig. 2b). Blots result in Fig. 2b show that SiHa and CaSki cells show two bands for STK31, this may be because that though the primary antibodies used for the western blot analyses were monoclonal anti-STK31, a nonspecific band was also detected. Analysis of the qPCR results revealed that STK31expression was induced to different levels in the C33A and HT-3 cells following treatment with different concentrations of 5-aza-dC (Fig. 2c). The expression of STK31 was induced and plateaued at different hours in the C33A and HT-3 cell lines when they were cultured in the optimum concentration of 10 μM 5-aza-dC (Fig. 2c). The results described above revealed that 5-aza-dC induced STK31 expression in CC cell lines and that this effect was concentration and time dependent.

### Clinicopathological significance of STK31 promoter/exon 1 methylation status in CC

The relations between the methylation status of STK31 and the clinicopathological characteristics in the patients with CC were analyzed. The hypomethylation status of STK31 was significantly associated with HPV16/18 infection, chemotherapy treatment and chemotherapy resistance (*p* < 0.05) and was not associated with age, the type of cancer, lymph node metastasis, cancer grade, the FIGO stage, tumor size, HPV genotyping or Squamous cell carcinoma antigen (SCC-Ag) level (*p* > 0.05; Table 1). Seventy patients with CIN3, including 49 cases with STK31 hypermethylation and 21 cases with STK31 hypomethylation, underwent histological follow-ups for 11 years. Invasive CC was found in 5 of 21 cases with STK31 hypomethylation (23.8 %) and in none of the 49 patients with STK31 hypermethylation (*p* < 0.05).

**Table 1:** Correlations between STK31 methylation and the different clinicopathological features of human CCs

Clinicopathological features	N	STK31 methylation		p
		+	-	
Age	148			
<45		49	13	0.741
≥45		66	20	
Histology	102			
Squamous cell carcinoma		67	13	1.000
Adenocarcinoma		18	4	
Differentiation	146			
Well + Moderate		75	17	0.584
Poor		42	12	
Lymph node metastasis	100			
Positive		23	7	0.708
Negative		56	14	
FIGO stage	119			
IA1 ~ IIA2		50	14	0.617
IIB ~ IIIA		45	10	
Tumor size (cm)	148			
<1		40	11	0.877
≥1		75	22	
HPV16/18	134			
No(HPV-negative)		18	0	0.037
Yes		87	29	
HPV genotyping	123			
HPV16		81	19	0.711
HPV18		20	3	
Squamous cell carcinoma-Ag(SCC-Ag)	80			
Normal		24	4	1.000
High		45	7	
Neoajuvant Chemotherapy	55			
No		23	7	0.029
Yes		25	0	
Neoajuvant Chemotherapy resistance	55			
No		44	9	0.037
Yes		0	2	

### Chemotherapy and STK31 expression

**Fig. 3:**
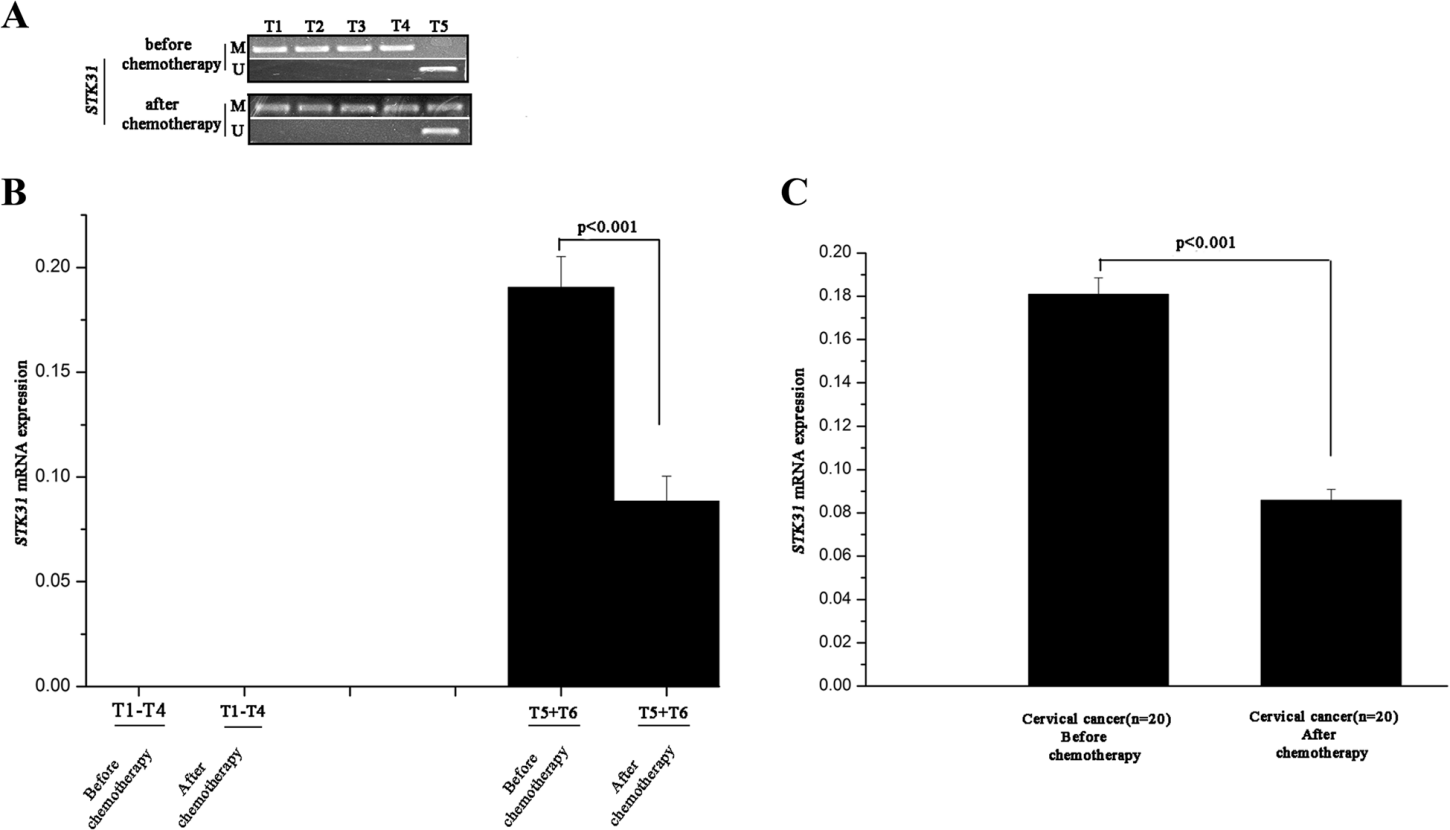
Chemotherapy decreased STK31 expression in cervial cancer by upregulating its promoter/exon 1 methylation status. **a** MSP results showed that the chemotherapy treatment could increase methylation status of STK31 in CC cases. **b** qPCR showed that chemotherapy could decrease STK31 expression in CC samples. **c** qPCR showing that expression of STK31 was higher in samples before neoadjuvant chemotherapy than the specimens after chemotherapy

The previously detailed results revealed that the STK31 was methylated to a greater extent in the CC patients who had undergone chemotherapy than in those who had not (Table 1, *p* < 0.05); thus, we assessed the correlation between chemotherapy and STK31 methylation status in the CC patients. The methylation status of the STK31 promoter/exon 1 was evaluated via the BGS and MSP methods in 10 HPV16 cervical patients before and after neoadjuvant chemotherapy. The BGS data revealed that 8 of the 10 CC patients exhibited nearly complete hypermethylation STK31 prior to neoadjuvant chemotherapy and exhibited no methylation changes after chemotherapy (T1-T4 are shown as examples of the cancer patients exhibited nearly complete hypermethylation STK31 prior to neoadjuvant chemotherapy and exhibited no methylation changes after chemotherapy), and 2 cases (T5-T6) of the 10 CC samples had statuses of hypomethylation prior to neoadjuvant chemotherapy and exhibited hypermethylation after chemotherapy (Additional file 3: Figure S3A-1). The MSP results (Fig. 3a) were consistent with the BGS findings. qPCR was applied to six of these CC cases (T1-T6) before and after neoadjuvant chemotherapy. The qPCR results revealed that no STK31 expression was detected cases T1-T4 before or after chemotherapy, while STK31 expression was markedly decreased in cases T5 and T6 following chemotherapy compared with the samples taken before neoadjuvant chemotherapy (Fig. 3b). The MSP method was then applied to select another 10 HPV16-postive CC patients with hypomethylation statuses before neoadjuvant chemotherapy. STK31 gene expression was measured with qPCR in these 10 MSP-selected patients before and after chemotherapy, and the results revealed that the mean expression of STK31 was higher in the samples before neoadjuvant chemotherapy than after chemotherapy (Fig. 3c).

## Discussion

The HPV oncoproteins E6 and E7 mediate the oncogenic activities of hrHPVs and are crucial for hrHPV-induced malignant cell transformation; furthermore these oncoproteins have been proven to affect human CC through different pathways [2, 3, 14, 15]. In the present study, we found that the HPV16 E7 oncoprotein induced the expression of STK31 in HPV-negative CC cells through an epigenetic mechanism, which indicates that STK31 may be a novel cellular target gene for the HPV16 oncogene E7. The restoration of STK31 expression by the demethylating agent 5-aza-dC in the C33A and HT-3 HPV-negative CC cell also supports the epigenetic role of STK31 in the etiology of cervical tumors. Due to the fact that the STK31 gene exerts its important effects in various human cancers via its biological functions of increasing cell migration and invasiveness, suppressing cell apoptosis, promoting tumorigenicity and maintaining the undifferentiated state of cancer cells [12, 13], it has been speculated that the inhibition of HPV oncogene E7 in HPV-positive CCs contributes to CC therapy by inhibiting the tumor gene STK31 activities. STK31 might therefore act as a potential therapeutic target in human CCs.

A small proportion of women with HPV infections that are left untreated develop invasive cervical carcinomas; therefore, the positive predictive value (PPV) of HPV testing for CC screening is very low. Additionally, there is no well-established national CC screening program in China [16, 17]. Therefore, the exploration of a panel of CC-specific biomarkers, e.g., DNA methylation biomarkers, with proven high sensitivities and specificities for cervical neoplasia [18–20] may be valuable for detecting cancer precursors early, reassuring candidates by reducing the frequency of screening, predicting the outcomes for women infected with hr-HPV, and guiding the treatment of HPV-related cervical tumors [19, 21]. Our previous studies (Fig. 2a) revealed that the regulation of the expression of STK31 by its methylation status is heterogeneous across the different stages of cervical lesions, which suggests that STK31 may be a precursor for human cervical carcinogenesis and is thus a potential DNA aberrant methylation biomarker for the early diagnosis of CC. The results of histological follow-ups showed that invasive CC was found in 5 of 21 (23.8 %) cases with STK31 hypomethylation and in none of the 49 patients with STK31 hypermethylation (*p* < 0.05), revealing the aberrant methylation of STK31in the patients with CIN3 may indicate a higher risk for the development of CC. Because the aberrant methylation of STK31 in the CIN3 biopsy cases indicated an increased possibility of developing into CCs, individual therapy may be needed and of great importance for such CIN3. Interestingly, CIN2 and CIN3 are classified together as high-grade intraepithelial lesions (HSIL), but our study found that the STK31 methylation in CIN2 is different from that in CIN3. Although CIN2 and CIN3 are HSILs, studies have shown some different indexes between CIN2 and CIN3 [22, 23], it’s necessary to detect the methylation status in CIN1, CIN2 and CIN3 respectively. Our results found that CIN3 represents a more advanced stage of intraepithelial neoplasia than CIN2, and the difference in STK31 methylation between CIN2 and CIN3 may be due to the different stages of the cervical tumors.

The development of chemotherapy resistance requires changes in the expressions of a number of genes. It has been hypothesized that epigenetic therapy holds the potential to reverse chemotherapy resistance [24]. A variety of genes have been proved to be associated with chemoresistance through different pathways [25–27]. The results of Table 1 showed that aberrant methylation of the STK31 gene was statistically correlated with resistance to chemotherapy. Results of Fig. 3 indicated that the expression patterns of STK31 before and after neoadjuvant chemotherapy were in accordance with the previously observed patterns of methylation changes, suggesting that chemotherapy may function in CC patients through an epigenetic mechanism. Nevertheless, the treatment of chemotherapy may result in extensively effects including signaling pathways, cell cycle regulation, apoptosis and metabolite, so further studies need to be focused on whether the altered methylation status was caused by the direct effect of chemotherapy treatment.

Based on the reversible nature of epigenetic aberrations, a number of DNA methylation and histone deacetylase (HDAC) inhibitors are being preclinically tested in combination for cancer therapy. These epigenetic therapeutic drugs have potential therapeutic effects due to abilities to exert synergistic effects on gene expression [28, 29] and tumor growth [30]. Recent studies have demonstrated that epigenetic drugs are able to increase the efficacy of chemoradiation and to reduce chemoresistance by inducing the demethylation activities of tumor suppressor genes and increasing their expressions [31–33]. Nevertheless, epigenetic therapies based on DNA methylation and histone deacetylase (HDAC) inhibitors are not suitable for all patients because the up-regulation of the expressions of oncogenes could render patients more resistant. The present study found that aberrant changes in the hypomethylation of STK31 were correlated with chemoresistance and the development of CC.

## Conclusions

In conclusion, though the processes that govern the degree of gene promoter methylation in HPV-induced carcinogenesis is quite complex, the present study primarily revealed that STK31 may be a novel cellular target gene for the HPV16 oncogene E7. The aberrant methylation of the STK31 promoter/exon 1 region may be a precursor for human cervical carcinogenesis and thus a potential DNA aberrant methylation biomarker of the progression of precancerous disease to invasive cancer. Overall, the STK31 gene represents a potential tool for the diagnoses, treatments and prognoses of CCs.

## Methods

### Cell lines and constructs

The HPV16/18-positive CC cell lines HeLa, CaSki, SiHa and the HPV-negative CC cells C33A and HT-3, all of which were obtained from Shanghai institute for biological sciences, Chinese academy of Sciences Institute of Cell Resource Center (Shanghai, China). In the present study, we established the ectopically expressed HPV16 E6, -E7, and -E6/E7 cell models of C33AE6, C33AE7, C33AE6/E7, HT-3E6, HT-3E7, and HT-3E6/E7 by transfecting HPV16 E6, -E7, and -E6/E7 oncogenes with lentivirus vectors into the HPV-negative CC cell line C33A and HT-3 cells in our lab. The C33A-vector (C33A-V) and HT-3 vector (HT-3 V) cells were established by transfecting C33A and HT-3 cells with lentivirus vectors that did not code for the HPV16 E6, -E7, or -E6/E7 proteins as controls. Stable transfectants were selected with 10 ug/ml puromycin for 3 weeks. The transfection efficiency was tested by western blotting, which revealed that the transfected cells successfully expressed the E6, -E7, or -E6/E7 proteins (Fig. 1a).

### Cell treatment with 5′-aza-2-deoxycytidine (5-aza-dC)

For the demethylation experiments, demethylation was induced with 5-aza-dC treatment at a concentration that was able to induce the demethylation of the DNA without killing the cells. HeLa, SiHa, CaSki, HT-3 and C33A cells were plated at a density of 8 × 10^4^ cells/25 cm^2^ flask and treated with various concentrations (5 μM and 10 μM) of 5-aza-dC for 96 h. We also cultured the five cells in a 10 μM 5-aza-dC medium for 0 h, 12 h, 24 h, 48 h, 72 h, 96 h and 120 h.

### Tissue specimens

This study was approved by the Institutional Review Board (IRB) of the Affiliated Hospital of Qingdao University. All human cervical samples, including 148 HPV16/18-positive and 18 HPV-negative CC samples that were without preoperative adjuvant chemotherapy (PACT), 55cases of HPV16/18-positive CC samples with PACT, 42 HPV16/18-positive normal cervical samples, 20 cases of HPV16/18-positive CIN1, 20 cases of HPV16/18-positive CIN2, 48 cases of HPV16/18-positive CIN3 were used in the study and were obtained with written informed consent from the donor who underwent primary surgical treatment for cervical tumors or other benigh uterine tumors from the Affiliated Hospital of Qingdao University between 2003 and 2015. All cases were reviewed by at least two pathologists to confirm the diagnoses. Paraffin blocks of the HPV16/18-positive CC biopsy samples before preoperative adjuvant chemotherapy (PACT), CIN1, CIN2, and CIN3 were taken from the Pathology Department of our hospital and processed under strict conditions to avoid contamination.

### HPV-DNA detection and genotyping

The detection of HPV DNA was performed by using HPV MY-PCR primers (MY09/MY11), GP5+/6+ and SPF1/2 methods. Both MY09/11 and GP5+/6+ were performed in a final reaction volume of 20 uL, containing 3 uL of the isolated DNA, 2 uL of the 10 × Buffer, 0.8 uL of each deoxynucleoside triphosphate, 0.4 uL of forward and reverse primers, and 1.0 U of Taq DNA polymerase (genebase bioscience, Guangzhou, China). PCR conditions of MY09/11 were as follows: preheating for 5 min at 94 °C was followed by 40 cycles of 1 30 s at 94 °C, 1 min at 45 °C, and 1 min at 72 °C and a final extension of 7 min at 72 °C; PCR conditions of GP5+/6+ were as follows: preheating for 10 min at 94 °C was followed by 40 cycles of 1 min at 94 °C, 2 min at 40 °C, and 1.5 min at 72 °C and a final extension of 7 min at 72 °C. Each PCR experiment was performed with positive and several negative PCR controls. The final PCR products were separated by electrophoresis in 1.8 % agarose gel. SPF was performed in a final reaction volume of 40 uL, containing 4 uL of the isolated DNA, 4 uL of the 10 × Buffer, 3.2 uL of each deoxynucleoside triphosphate, 0.5 uL of forward and reverse primers, and 2.5 U of Taq DNA polymerase (genebase bioscience, Guangzhou, China). PCR conditions were as follows: preheating for 1 min at 94 °C was followed by 40 cycles of 1 min at 94 °C, 1 min at 45 °C, and 1 min at 72 °C and a final extension of 5 min at 72 °C. Each PCR experiment was performed with positive and several negative PCR controls. The products of PCR were then fractionated on 12 % polyacrylamide gel electrophoresis, PAGE gels. As for all methods of HPV-DNA detection and genotyping, see Table 2 for the detailed primer sequences and size of the product. HPV-detection results of the three methods were all consistent with each other, the negtive ones were demonstrated as HPV-negative samples and the positive samples were further etected by HPV type specific PCR for HPV 16 and HPV 18 [34]. Details of PCR are available from the corresponding author.

**Table 2:** The primer sequences and product size of MY09/11, GP5+/6+ and SPF1/2

Primer	sequence	Size (bp)
MY09/11	F: 5ʹ- CGTCCMARRGGAWACTGATC- 3ʹ	450
	R: 5ʹ- GCMCAGGGWCATAAYAATGG- 3ʹ	
GP5+/6+	F: 5ʹ- TTTGTTACTGTGGTAGATACTAC- 3ʹ	150
	R: 5ʹ- GAAAATAAACTGTAAATCATATTC - 3ʹ	
SPF1A	F: 5ʹ- GCiCAGGGiCACAATAATGG - 3ʹ	65
SPF1B	R: 5ʹ- GCiCAGGGiCATAACAATGG - 3ʹ	
SPF1C	F: 5′- GCiCAGGGiCATAATAATGG - 3′	
SPF1D	R: 5′- GCiCAAGGiCATAATAATGG - 3′	
SPF2B-bio	F: 5′-GTiGTATCiACAACAGTAACAAA - 3′	
SPF2D-bio	F: 5′-GTiGTATCiACTACAGTAACAAA- 3′	

### RNA extraction and cDNA synthesis

Total RNA was isolated from the CC cells and cervical tissues using the Trizol reagent (Invitrogen, Carlsbad, CA, USA). A RNeasy FFPE Kit (QIAGEN GmbH, Hilden, Germany) was used to extract the RNA from paraffin-embedded cervical tissues. One microgram of RNA was used to obtain cDNA using the Transcriptor First Strand cDNA Synthesis Kit (Roche, Mannheim, Germany) according to the manufacturer’s protocol. The cDNAs were then used for reverse-transcription PCR (RT-PCR) and quantitative real-time PCR (qPCR).

### RT-PCR and qPCR

Conventional RT-PCR and qPCR were performed to examine the expression of the STK31 gene. A PCR assay with the specific primers for GAPDH cDNA was performed for each sample to ensure the integrity of the RNA. The final PCR products were separated by electrophoresis in 2 % agarose gel. See Table 3 for the primer sequences and PCR conditions.

**Table 3:** Primer sequences and reaction conditions for the BGS, MSP and RT-PCR experiments

Primer	sequence	Size (bp)	Temperature (°C)
STK31-BGS	F: 5ʹ-TTTTTAAAGTTATAGTTTGAAGTTTTG- 3ʹ	499	50
	R: 5ʹ- ACATCTAACACCCCTCTAAAATAAC - 3ʹ		
STK31-M	F: 5ʹ- TTGTTACGTGATTTTCGTTAATATC - 3ʹ	149	51
	R: 5ʹ-TAAACCCACATACTAAACTTTCGAC - 3ʹ		
STK31-U	F: 5ʹ-GTTATGTGATTTTTGTTAATATT - 3ʹ	147	51
	R: 5ʹ-TAAACCCACATACTAAACTTTCAAC - 3ʹ		
STK31-RT	F: 5′-AGAGGAATATGAGATGCTAACTA - 3′	111	60
	R: 5′- GTAAGGAGACCACCAGAG - 3′		
GAPDH	F: 5′- GATGACCTTGCCCACAGCCT - 3′	303	60
	R: 5′- ATCTCTGCCCCCTCTGCTGA - 3′		

### DNA extraction and bisulfite genomic sequencing (BGS)

The DNA was extracted by using SDS-proteinase K and purified with phenol:chloroform:isoamyl alcohol. A QIAampDNAFFPE tissue kit (QIAGENGmbH, Hilden, Germany) was used of the DNA extraction from the paraffin-embedded tumor tissues. The bisulfite conversion of the DNA was performed using an EpiTect Fast DNA Bisulfite kit (Qiagen, Valencia, CA, USA) according to the instruction of the manufacturer. The methylation statuses of the promoter/exon 1 region of STK31 in the human CC cells and cervical specimens were analyzed by BGS. The primer sequences and conditions for the BGS are presented in Table 3. The purified PCR products were cloned into the pUC18-T vector, and six clones from each sample were randomly selected and sequenced.

### Methylation-specific PCR (MSP)

MSP was performed to test the methylation status of the STK31 gene promoter/exon 1 region. The specific primer pairs and PCR conditions are listed in Table 3.

### Western blotting

The harvested cells were lysed in RIPA lysis buffer supplemented with protease inhibitor cocktail. Each protein lysate (30 ug) was mixed with 5× SDS-PAGE sample loading buffer, and the mixtures were boiled for 5 min. The boiled mixtures (50 μg) were then fractionated on 12 % SDS-PAGE gels and subsequently transferred onto PVDF membranes (Merck Millipore, Billerica, MA, USA). The primary antibodies used for the western blot analyses were anti-STK31 and anti-β-actin. β-actin was used as a housekeeping protein to normalize the protein loads.

### Statistical analysis

The comparison of the STK31 expressions between the groups were performed with a Student’s *t*-test. The correlations between the clinicopathological characteristics and the numbers of methylated specimens were tested using the *χ*2-test. Differences were considered significant at *p* < 0.05, and all statistical tests were performed with SPSS version 17.0 (SPSS, Chicago, USA).

## Additional files

Additional file 1: Figure S1D-1. BGS analyses of CC cells before and after being transfected with HPV16 E6 or/and E7. (TIF 861 kb) Additional file 2: Figure S2B-1. Results of BGS in CC cell lines before and after being treated with 5-aza-dC. (TIF 726 kb) Additional file 3: Figure S3A-1. BGS results of CC simples before and after being treated with chemotherapy. (TIF 352 kb)

## Abbreviations

STK31: Serine/threonine kinases 31
hrHPV: high-risk human papillomavirus
CT: cancer/testis
BGS: Bisulfite genomic sequencing PCR
MSP: methylation-specific PCR
CIN: cervical intraepithelial neoplasia
5-aza-dC: 5′-aza-2-deoxycytidine
PPV: positive predictive value
HSIL: high-grade intraepithelial lesions
HDAC: histone deacetylase
IRB: Institutional Review Board
PACT: preoperative adjuvant chemotherapy
