# Supplementary figures and images for: Serine/threonine kinases 31(STK31) may be a novel cellular target gene for the HPV16 oncogene E7 with potential as a DNA hypomethylation biomarker in cervical cancer
Source: Virol J. 2016 Apr 5;13:60. doi: 10.1186/s12985-016-0515-5 (PMC4820863; doi:10.1186/s12985-016-0515-5)

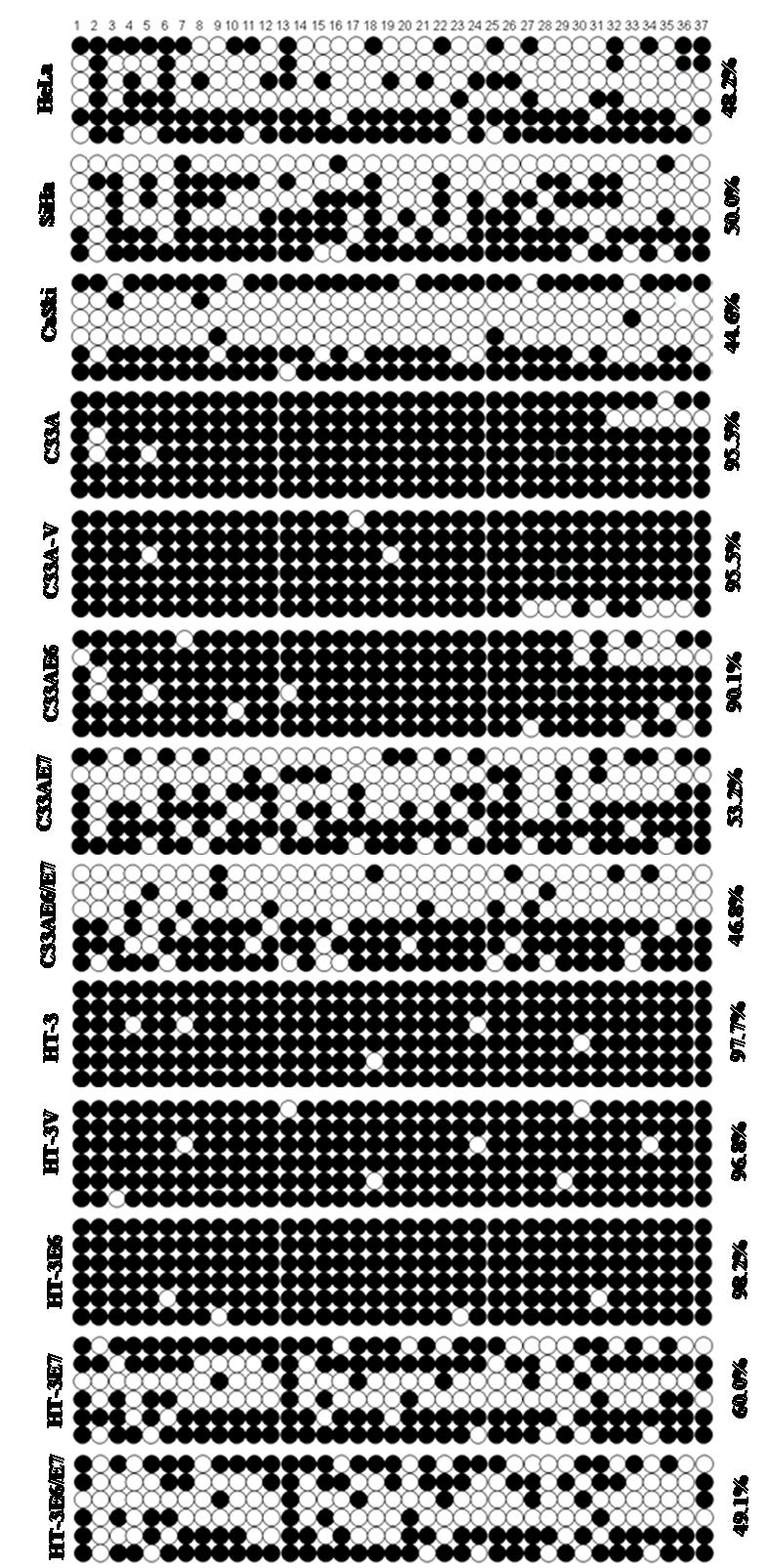

Supplement: Additional file 1: Figure S1D-1. — BGS analyses of CC cells before and after being transfected with HPV16 E6 or/and E7. (TIF 861 kb) [file 12985_2016_515_MOESM1_ESM.tif]

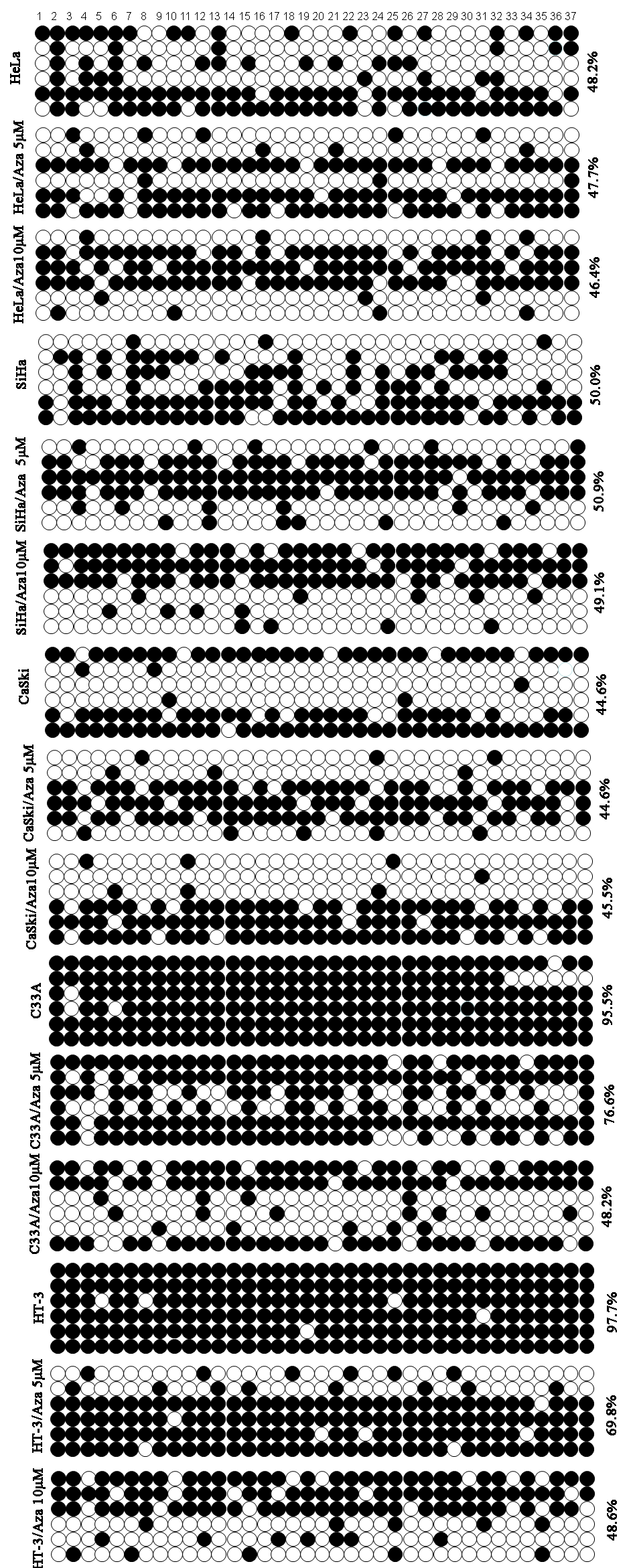

Supplement: Additional file 2: Figure S2B-1. — Results of BGS in CC cell lines before and after being treated with 5-aza-dC. (TIF 726 kb) [file 12985_2016_515_MOESM2_ESM.tif]

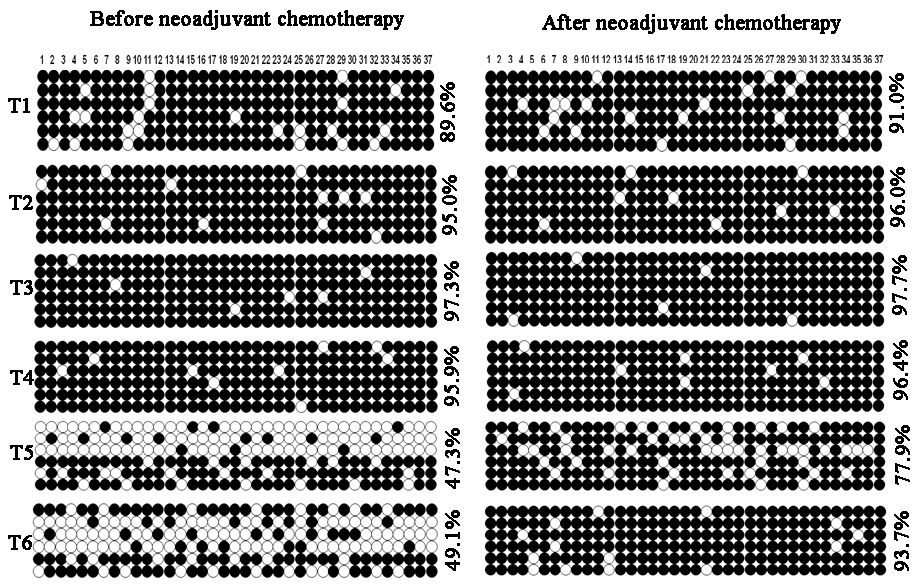

Supplement: Additional file 3: Figure S3A-1. — BGS results of CC simples before and after being treated with chemotherapy. (TIF 352 kb) [file 12985_2016_515_MOESM3_ESM.tif]
